# Efficacy of ICON^® ^Maxx in the laboratory and against insecticide-resistant *Anopheles gambiae *in central Côte d'Ivoire

**DOI:** 10.1186/1475-2875-11-167

**Published:** 2012-05-15

**Authors:** Mirko S Winkler, Emile Tchicaya, Benjamin G Koudou, Jennifer Donzé, Christian Nsanzabana, Pie Müller, Akré M Adja, Jürg Utzinger

**Affiliations:** 1 Department of Epidemiology and Public Health, Swiss Tropical and Public Health Institute, P.O. Box, CH-4002 Basel, Switzerland; 2 University of Basel, P.O. Box, CH-4003 Basel, Switzerland; 3 Département Environnement et Santé, Centre Suisse de Recherches Scientifiques en Côte d'Ivoire, 01 BP 1303 Abidjan 01, Côte d'Ivoire; 4 UFR Biosciences, Université de Cocody, 22 BP 582 Abidjan 22, Côte d'Ivoire; 5 UFR Sciences de la Nature, Université d'Abobo-Adjame, 02 BP 801 Abidjan 02, Côte d'Ivoire; 6 Vector Group, Liverpool School of Tropical Medicine, Pembroke Place, Liverpool L3 5QA, UK; 7 Department of Parasitology, University of Neuchâtel, P.O. Box, CH-2000 Neuchâtel, Switzerland; 8 Department of Medical Services and Diagnostic, Swiss Tropical and Public Health Institute, P.O. Box, CH-4002 Basel, Switzerland; 9 Institut Pierre-Richet, 01 BP 1500, Abidjan 01, Côte d'Ivoire

## Abstract

**Background:**

Long-lasting treatment kits, designed to transform untreated nets into long-lasting insecticidal nets (LLINs), may facilitate high coverage with LLINs where non-treated nets are in place. In this study, the efficacy of ICON^® ^Maxx (Syngenta) was evaluated under laboratory conditions and in an experimental hut trial in central Côte d'Ivoire, where *Anopheles gambiae s.s*. are resistant to pyrethroid insecticides.

**Methods:**

In the laboratory, polyester and polyethylene net samples were treated with ICON^® ^Maxx, washed up to 20 times and their efficacy determined in World Health Organization (WHO) cone assays against a susceptible laboratory *An. gambiae s.s*. colony. Over a 12-month period, the polyester nets were evaluated in a hut trial to determine mosquito deterrence, induced exophily, blood-feeding inhibition and mortality.

**Results:**

In the laboratory, ICON^® ^Maxx-treated polyethylene nets showed higher efficacy against pyrethroid-susceptible mosquitoes than polyester nets. After 20 washings, insecticidal efficacy in bioassays was 59.4% knockdown (KD) and 22.3% mortality for polyethylene, and 55.3% KD and 17.9% mortality for polyester nets. In experimental huts, treated nets showed strong deterrence, induced exophily and an over three-fold reduction in blood-fed mosquitoes. More than half (61.8%) of the mosquitoes entering the huts with treated nets were found dead the next morning despite high levels of KD resistance. After washing the treated nets, KD and mortality rates were close to or exceeded predefined WHO thresholds in cone bioassays.

**Conclusion:**

In contrast to previous laboratory investigation, ICON^® ^Maxx-treated nets showed only moderate KD and mortality rates. However, under semi-field conditions, in an area where mosquitoes are resistant to pyrethroids, ICON^® ^Maxx showed high deterrence, induced exophily and provided a significant reduction in blood-feeding rates; features that are likely to have a positive impact in reducing malaria transmission. The WHO cone test may not always be a good proxy for predicting product performance under field conditions.

## Background

Insecticide-treated nets (ITNs) are an efficacious and cost-effective measure to reduce malaria morbidity and mortality [1-3]. As conventional ITNs need to be re-treated with insecticide at least once a year, or after two or three washes, their effective protection in the long-term may be compromised [4]. To overcome this constraint, manufacturers have developed long-lasting insecticidal nets (LLINs). Insecticides remain present, either incorporated into, or coated around, the fibre at toxic concentrations for malaria vectors even after multiple washes. Biological activity then lasts for several years with no need for repeated re-treatment [5-10]. Hence, LLINs have become an important tool for vector control against malaria and other mosquito-borne diseases [11-13]. There is a growing demand for LLINs within the frame of national malaria control programmes, so that the target of at least 85% of the at-risk populations to sleep under an ITN by 2015 can be reached [14,15].

Progress has also been made with long-lasting treatment kits that transform untreated nets into LLINs after simple dipping, combining a conventional insecticide with a binding agent. Untreated and conventionally-treated nets already in use could be transformed into LLINs by applying a long-lasting treatment kit. Currently, the only long-lasting insecticide treatment kit with recommendations from the World Health Organization Pesticide Evaluation Scheme (WHOPES) is ICON^® ^Maxx, developed by Syngenta and released to the market in 2007 [16,17].

In this study, the efficacy of ICON^® ^Maxx was evaluated under tropical conditions in Côte d'Ivoire. The efficacy and wash resistance were first assessed in the laboratory on two different net types following the WHOPES guidelines [18]. Subsequently, ICON^® ^Maxx was tested under semi-field conditions in experimental huts in central Côte d'Ivoire during a 12-month period, following a case-control design, with half of the nets washed once after six months. Of note, the main objective of this part of the study was to determine the performance of ICON^® ^Maxx under 'real-life' conditions, and hence WHOPES guidelines were not strictly followed. The study was implemented in central Côte d'Ivoire, where *Anopheles gambiae s.s*. are resistant to pyrethroids [19,20].

## Methods

### ICON^® ^Maxx treatment kit

ICON^® ^Maxx is based on a slow release 10% capsule suspension formulation of the pyrethroid lambda-cyhalothrin, combined with a polymer binding agent. The binding agent retains the capsules on the fibre during washing and the capsule suspension technology allows a controlled release of insecticide over time. Each ICON^® ^Maxx treatment kit contains one sachet of micro-encapsulated lambda-cyhalothrin ICON^® ^10CS, one sachet of binding agent, treatment instructions, one pair of disposable gloves and a water-measuring bag. One kit is designated for the post-manufacture treatment of one net and is applicable to nets of multiple sizes.

### Laboratory investigation

#### Net treatment

The laboratory evaluation was carried out on two different netting materials widely used in Côte d'Ivoire, polyester and polyethylene. Nets were purchased from a local market in Abidjan in September 2007 (colour: white; no further specifications available). Four nets of each type, plus an additional one serving as control, were treated with ICON^® ^Maxx diluted in deionized water and dried in the shade following the manufacturer's instructions. Net samples were cut into square pieces of 25 × 25 cm. From each net, six samples were sliced at randomly selected positions (samples were scheduled for 0, 1, 5, 10, 15 and 20 washings).

For the washing procedure, net samples were individually introduced into 1 l plastic bottles containing 500 ml deionized water, with 1 g of previously dissolved soap ('Savon de Marseille' produced by Unilever). Bottles were placed in a water bath at 30°C and shaken for 10 min at 155 movements per min. Next, the samples were removed and rinsed twice for 10 min under the same conditions as stated above. Nets were dried in the shade and stored in plastic bags in a cupboard at room temperature. Between the washings, a 24 h interval was respected.

#### Mosquitoes

*Anopheles gambiae s.s*. Kisumu eggs were sent from the Institut Pierre Richet (IPR) in Bouaké, central Côte d'Ivoire, on a wettish blotting paper, directly to the insect rearing room at Abidjan, where they were introduced in a vessel half filled with deionized water. Each vessel was provided a diet of lab chow (Tetra^® ^Mikromin; Spectrum Brands Inc., Germany) once daily. When the nymph-stadium was reached, nymphs were transferred to a 100 ml plastic beaker using a pipette. The beaker was introduced into a mosquito cage (30 × 30 × 30 cm) together with cotton pads soaked with a 10% honey solution. Cages were annotated with the actual date and mosquitoes were used for testing at the latest on the fifth day after introduction of the beaker. Cotton wool-pellets imbued with 10% honey solution were changed daily.

For stock authentication of *An. gambiae s.s*. Kisumu strain, a sub-sample of 40 adult females were screened against presence of the L1014S and L1014F *kdr *allele according to the procedure developed by Martinez-Torres et al. (1998) [21].

#### Bioassays

Efficacy of ICON^® ^Maxx was assessed in WHO cone assays [18] using non-blood-fed, two-to five-day-old adult females from a susceptible *An. gambiae s.s*. Kisumu strain established at IPR for each of the two netting types (i.e. polyethylene and polyester). The assays were performed with unwashed and then repeated with washed netting material after 1, 5, 10, 15 and 20 washings. The implementation of the bio-efficacy test included four treated net samples and one control. For each treated net, 50 mosquitoes were tested in 10 cone bioassays i.e. five mosquitoes per cone.

In cone bioassays, five mosquitoes were introduced into a cone at a time and removed to a plastic beaker after 3 min of exposure at an angle of approximately 45°, using a mouth aspirator. Knock-down (KD) (i.e. mosquitoes are not moving anymore when gently knocking the beaker) was measured at the end of the exposure time (3 min) and again 60 min post-exposure. After 3 min, mosquitoes were transferred to holding containers with access to a 10% honey solution. Mortality was recorded 24 h after exposure. Mosquitoes that had lost at least three legs or at least one wing were deemed dead. Bioassays were carried out at a temperature of 25 ± 2°C and a relative humidity of 75 ± 10%.

### Experimental hut trial

#### Study area and experimental set-up

The efficacy of nets treated with ICON^® ^Maxx was evaluated over a 12-month period (June 2008 to May 2009) in experimental huts at M'Bé station, 35 km north-west of Bouaké, central Côte d'Ivoire (geographical coordinates: 7°53'45'' N latitude and 5°05'51'' W longitude) [22,23]. In this area a recent study found high frequency of *kdr *in pyrethroid-resistant *An. gambiae s.s*. with high frequency of heterozygotes (97.3% resistant heterozygote) and low frequency of homozygotes (2.7% resistant homozygotes) [24]. Additionally, the species and molecular forms of the *An. gambiae *complex were identified in the study area using a PCR method described by Fanello *et al *[25]. *Anopheles gambiae *S was the predominant form (92%) with a low rate of *An. gambiae *M form (8%).

Four experimental huts were used, following a case-control design. Two of the huts were equipped with ICON^® ^Maxx-treated polyester nets (most common net type in Côte d'Ivoire), whereas the remaining huts were equipped with untreated nets of the same type that served as control. According to WHOPES guidelines [18], experimental huts were fitted with entry slots, exit traps and screened verandas.

#### Volunteer sleepers, rotation and mosquito collection

Adult volunteer sleepers (males, aged 18-40 years) spent six nights peer week (from 20:00 to 06:00 hours) under the mosquito nets in the experimental huts [26]. Sleepers were rotated among the huts every night of the study. Bedding and pyjamas did not rotate to avoid insecticide contamination of the controls. Once every week, the huts were cleaned and thoroughly aired to avoid contamination.

Each morning, collected mosquitoes were transferred to the laboratory of IPR and identified to the genus and species level using a readily available determination key [27]. Mosquitoes were classified as (i) dead/blood-fed; (ii) alive/blood-fed; (iii) dead/unfed; and (iv) alive/unfed. Surviving mosquitoes were provided with a 10% honey solution and kept for 24 h after which delayed mortality was determined. For *An. gambiae *and *An. funestus*, the following parameters were assessed: (i) deterrent effect (reduction in hut entry relative to the control huts); (ii) induced exophily (proportion of mosquitoes found in the exit traps); (iii) blood-feeding rate; and (iv) immediate (in the morning) and delayed (24 h after collection) mortality rates. All parameters were reported for each hut separately.

After six months and in keeping with local practices, two of the nets (one treated and an untreated control net) were removed from the huts and washed separately using tap water and 'Savon de Marseille' type soap. The manual washing procedure, performed by women living in the study area, took approximately 10 min per net. Subsequently, the nets were rinsed twice and then dried in the shade.

Furthermore, five cone bioassays (one on each side of the rectangular-shaped net and one on top) were carried out at the end of each month on each net according to the procedure described above, with the only difference that 10 rather than five mosquitoes were introduced in a cone at a time. KD and mortality rates were determined.

### Ethical considerations

Ethical approval was obtained from the Ministry of Health in Côte d'Ivoire, through the national malaria control programme. Volunteer sleepers provided oral informed consent, as most of them were illiterate. The volunteer sleepers were offered free anti-malarial chemotherapy (artesunate plus amodiaquine, according to national policies), and their vaccination status against yellow fever was checked before enrolment. Medical supervision was provided throughout the study and for an additional six months after the end of the study by qualified medical personnel.

### Statistical analysis

Statistical analysis was performed in the open source software package R version 2.14.1 http://www.r-project.org using the "lme4" library for linear mixed-effects models [28]. For statistical testing the level of significance was set at *α *= 0.05.

Data obtained from mosquitoes collected in the experimental huts were analysed for four groups, (i) *An. gambiae s.s*.; (ii) *An. funestus*; (iii) other *Anopheles *species; and (iv) all other mosquitoes not included in the first three groups. For each of the four groups the following outcomes were compared between treated and untreated nets: (i) deterrence (i.e. reduction in hut entry relative to the control); (ii) induced exophily (i.e. the proportion of mosquitoes that were found in the exit traps); (iii) blood-feeding inhibition (i.e. the reduction of blood-feeding compared with that in the control huts); and (iv) mortality (i.e. the proportion of mosquitoes that were killed either immediately or 24 h post-collection. Deterrence was analysed as the reduction in total numbers (sum of mosquitoes caught in room and veranda) using a Poisson generalized linear mixed model (GLMM) with a log link function. For the proportional outcomes induced exophily, blood-feeding inhibition and mortality, the Poisson GLMM was replaced by a binomial generalized linear model (GLM) with a logit link function. The model terms for the fixed effects were *SPECIES *+ *SPECIES:TREATMENT*, where *SPECIES *is a factor for the different mosquito species groups and *SPECIES:TREATMENT *an interaction term for the species (i.e. mosquito species group) specific treatment effect. The model terms for the random effects were *DAY*, *SLEEPER *and *ROW*. While *DAY *accounted for the temporal random effects, *SLEEPER *accounted for the random variation between sleepers. The random effect *ROW *accounted for over-dispersion of the data and assigns a random effect for each observation (i.e. data row in the data table; hence the label *ROW*). For reporting, the relative rates (RR) for the aforementioned outcomes are given with their respective 95% confidence intervals (CI) for each of the four groups of mosquitoes.

## Results

### Findings from laboratory investigations

#### Baseline KD mortality rates

Prior to washing, the baseline KD and mortality rates of *Anopheles *for polyester and polyethylene nets treated with ICON^® ^Maxx were measured. Table 1 shows the mean percentage of KD and dead mosquitoes, as determined by the 3 min exposure efficacy tests. KD was measured 60 min post-exposure. Means of each net-type are based on the exposure of 200 mosquitoes to four treated net samples. None of the treated nets showed 100% baseline mortality, but with a KD of 98.0%, ICON^® ^Maxx was highly efficacious when applied on polyethylene nets. KDs for all untreated control net samples were 0%.

**Table 1:** Mean KD/mortality 60 min/24 h post-exposure to polyester and polyethylene nets after treatment with ICON^® ^Maxx under laboratory conditions in Côte d'Ivoire

Time post-exposure	Outcome measure	*An. gambiae *Kisumu	
		
		Polyester	Polyethylene
60 min	Mean KD	88.7%	98.0%
	(95% CI)	(82.2-95.2%)	(95.4-100.0%)
	[range]	[84.3-94.2%]	[96.0-100.0%]
24 h	Mean mortality	52.5%	45.7%
	(95% CI)	(38.9-66.1%)	(19.27-72.0%)
	[range]	[42.3-61.5%]	[30.6-68.0%]

#### KD and mortality rates after washing

**Figure 1:**
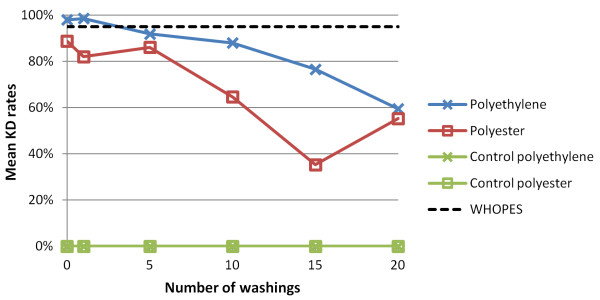
**The effect of washings on the mean KD of *An. gambiae *Kisumu-susceptible strain after 3 min bioassay test on polyester and polyethylene nets, measured 60 min post-exposure**.

Mean KD rates of *An. gambiae *Kisumu exposed to the net samples after 0, 1, 5, 10, 15 and 20 washings are shown in Figure 1. Both net types showed a clear decrease from 98.0% (polyethylene) and 88.7% (polyester) for the unwashed samples to 59.4% and 55.3% after 20 washings, respectively. Hence, after 20 washings, KD rates were considerably below the mean KD defined for LLINs by WHOPES guideline (i.e. 95% after 20 washings). The polyester net showed higher fluctuation in loss of efficacy than polyethylene nets. The untreated control net, washed the same way as the treated ones, revealed a constant KD of 0%.

**Figure 2:**
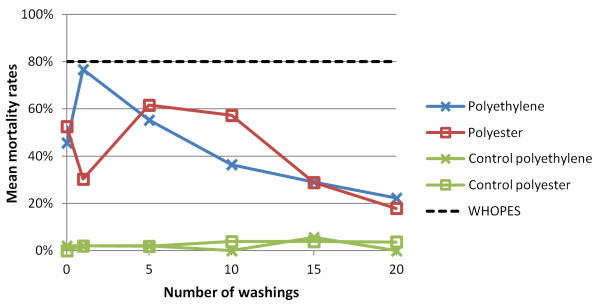
**The effect of washings on the mean mortality of *An. gambiae *Kisumu-susceptible strain after 3 min bioassay test on polyester and polyethylene nets, measured 24 h post-exposure**.

Mosquito mortality rates 24 h post-exposure are presented in Figure 2. There was considerable variation in mortality of the different treated net samples with the same number of washings. After one washing, polyethylene peaked with a mean mortality of 76.7%, whereas polyester showed the lowest value (30.3%). Overall the mean mortality decreased for both netting materials. After 20 washings polyester was 30.2% less lethal for exposed mosquitoes when compared with the baseline estimate. The respective reduction for polyethylene was 23.4%.

#### Kdr mutation in An. gambiae Kisumu strain

On the gel electrophoresis-plot obtained with the PCR-diagnostic test for identification of the *kdr *allele, the susceptible band (137 bp) was visible, the resistant band (195 bp) was missing and the common band existed. Hence, the genotype of *An. gambiae *Kisumu strain was SS: homozygous, standard susceptible [21].

### Results from the experimental hut trial

#### Mosquito abundance

From June 2008 to May 2009, a total of 17,373 mosquitoes were sampled by the four volunteer sleepers based on 1,176 man-nights of collection (Table 2). On average, 1,448 mosquitoes were caught per month, with a maximum of 1,860 mosquitoes in April 2009 and a minimum of 705 mosquitoes in January 2009. *An. gambiae *was the predominant species (62.9%). *An. funestus *accounted for 7.1% and other *Anopheles *species for 2.2%. The remaining 27.8% of mosquitoes belonged to other genera (i.e. *Culex*, *Aedes *and *Mansonia*).

**Table 2:** Average numbers and rates of the 12-month experimental hut trial at M'Bé station, in central Côte d'Ivoire, including numbers of entering and trapped mosquitoes, and mortality and blood-feeding rates

		Outcomes			
		
	Total number	Mosquitoes caught in exit traps*	Blood-feeding rate**	Immediate mortality rate***	Delayed mortality rate****
***An. gambiae *(n = 10,922)**					

Treated huts	2,816 (25.9%)	73.8%	4.9%	49.5%	9.4%

Control huts	8,106 (74.1%)	44.8%	13.6%	13.2%	2.4%

***An. funestus *(n = 1,234)**					

Treated huts	140 (10.9%)	60.7%	3.6%	66.4%	5.0%

Control huts	1,094 (89.1%)	34.8%	12.7%	10.2%	5.0%

**Other *Anopheles *(n = 384)**					

Treated huts	92 (21.5%)	47.8%	7.6%	78.3%	3.3%

Control huts	295 (78.5%)	50.8%	23.4%	22.7%	4.7%

**Other species (n = 4,830)**					

Treated huts	1,541 (31.4%)	44.7%	0.3%	82.9%	0.9%

Control huts	3,289 (68.2%)	41.7%	7.0%	10.9%	0.6%

**All mosquitoes (n = 17,373)**					

Treated huts	**4,589 (26.4%)**	**62.7%**	**3.4%**	**61.8%**	**6.3%**

Control huts	**12,784 (73.6%)**	**43.3%**	**12.0%**	**12.6%**	**2.2%**

* Percentage of mosquitoes caught in exit traps = total # mosquitoes caught in exit traps/total

# mosquitoes entering the hut * 100

** Blood-feeding rate = total # blood-fed mosquitoes/total # mosquitoes entering * 100

*** Immediate mortality rate = total # mosquitoes death mosquitoes in the morning/total # mosquitoes entering the hut * 100

**** Delayed mortality rate = total # mosquitoes death mosquitoes 24 h after collection/total # mosquitoes entering the hut * 100

#### Deterrence rate

Altogether, three-quarter (73.6%) of the total number of mosquitoes were caught in the control huts, and hence there was a considerable deterrent effect of ICON^® ^Maxx. Deterrence was highest on *Anopheles *spp. (RR = 0.33; *p *< 0.001) with, on average, 75.7% of the mosquitoes entering the control huts, compared to 68.1% of the other genera.

#### Induced exophily rate

Most of the mosquitoes entering the treated huts (62.7%) were caught in the exit traps (induced exophily). The respective percentage of mosquitoes that entered the control huts was 43.7%. *An. gambiae s.s*. induced exophily recorded in the treated huts was significantly higher than the one recorded in the control huts (RR = 4.31, 95% CI: 3.56-5.22) (Table 3). Similar results were observed for *An. funestus *(RR = 3.71, 95% CI: 2.29-6.03). In the treated huts, induced exophily was highest for *An. gambiae *(73.8% of the mosquitoes were caught in the exit traps). For species other than *Anopheles*, exophily was similar for the treated and the control huts (44.8% *versus *41.7%).

**Table 3:** Relative rates of outcomes from treatment compared to control

		Relative rate (95% confidence interval)		
	
Mosquito group	Deterrence (95% CI)	Induced exophily (95% CI)	Blood-feeding inhibition (95% CI)	Mortality (95% CI)
*An. gambiae s.s*.	0.33 (0.28-0.39)**	4.31 (3.56-5.22)**	0.27 (0.19-0.37)**	8.8 (7.4-10.4)**
*An. funestus*	0.12 (0.09-0.16)**	3.71 (2.29-6.03)**	0.15 (0.05-0.50)*	18.5 (11.6-29.7)**
*Anopheles *spp.	0.29 (0.22-0.40)**	0.91 (0.51-1.61) n.s.	0.24 (0.08-0.66)*	17.2 (9.0-32.9)**
Other	0.43 (0.37-0.52)**	1.12 (0.89-1.41) n.s.	0.04 (0.01-0.12)**	60.8 (47.2-78.5)**

n.s. = not significant, * *p *< 0.01, ** *p *< 0.001

#### Blood-feeding rate

**Figure 3:**
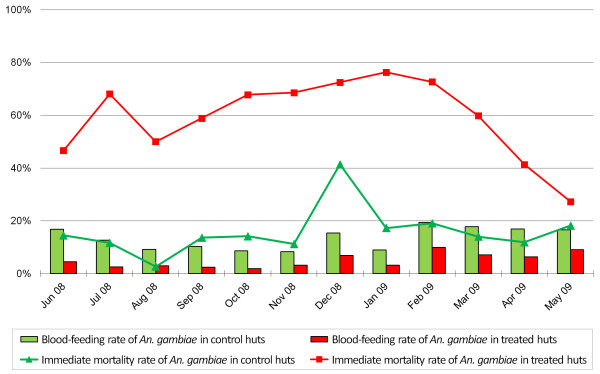
**Mortality rate and blood-feeding rate in ICON^® ^Maxx-treated huts *versus *control huts during a 12-month experimental hut trial in the M'Bé station, central Côte d'Ivoire**.

On average, the portion of blood-fed mosquitoes was several-fold higher in the control huts (12.0%) than in the treated huts (3.4%). The blood-feeding rate was lowest for species other than *Anopheles *with 0.3% for treated huts and 7.0% for controls. *An. gambiae *showed a mean blood-feeding rate of 4.9% in treated huts and 13.6% in the controls, with a slight increase towards the end of the experimental hut trial, as shown in Figure 3.

The *An. gambiae s.s*. blood-feeding rate recorded in the control huts was higher than the one recorded in the treated huts (RR = 0.27, 95% CI: 0.19-0.37). Similar results were observed for *An. funestus *(RR = 0.15, 95% CI: 0.05-0.50) and species other than *Anopheles *(RR = 0.04, 95% CI: 0.01-0.12).

#### Mortality rate

In the morning, 61.8% of the mosquitoes sampled in the treated huts were dead (immediate mortality), compared to 12.6% in the control huts. For *An. gambiae*, immediate mortality reached the highest level (76.3%) after eight months (January 2009), followed by a steady decrease to 27.3% (Figure 3). Overall, delayed mortality rates (24 h after collection) were higher for *An. gambiae *(treated huts: 9.4% and control huts: 2.7%) than for species others than *Anopheles *(treated huts: 0.9% and control huts: 0.6%).

#### Mean KD and mortality rate in cone bioassays

**Figure 4:**
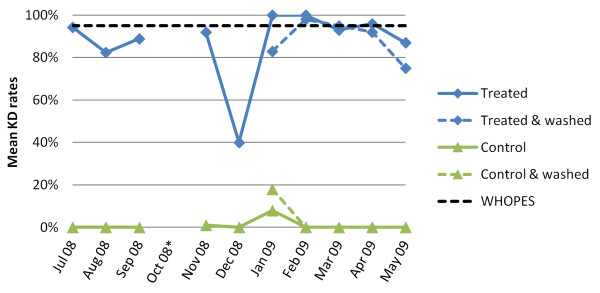
Development of mean KD (60 min post-exposure) after 3 min bioassay test for *An. gambiae *Kisumu-susceptible strain over a 12-month experimental hut trial in the M'Bé station, central Côte d'Ivoire, including the effect of the washings on treated an control nets (* data omitted due to insufficient number of mosquitoes).

**Figure 5:**
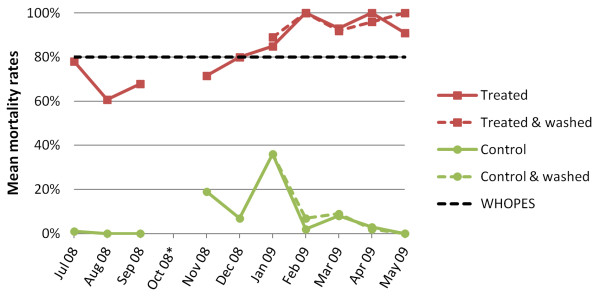
Development of mean mortality rates (24 h post-exposure) after 3 min bioassay test for *An. gambiae *Kisumu-susceptible strain over a 12-month experimental hut trial in the M'Bé station, central Côte d'Ivoire, including the effect of the washings on treated an control nets (*data omitted due to insufficient number of mosquitoes).

The dynamics of the mean KD and mortality rates of the cone bioassay efficacy test are shown in Figures 4 and 5. Over the 12-month experimental hut trial period, mean KD of *An. gambiae *Kisumu exposed to the treated nets remained at relatively high levels with a trend of higher KDs in the second half of the trial following net washing. Mean KDs for untreated net samples that served as controls were very low, with a small peak in January 2009 (unwashed control: 8.0%; washed control: 18.0%).

The mean mortality rate of *An. gambiae *Kisumu exposed to the treated nets showed a decrease in the second month from 78.1% to 60.8%. Subsequently, the mean mortality increased to a level above 80%, hence above the predefined WHOPES threshold for the second half of the experimental hut trial (Figure 5), including two months with 100% mean mortality. For the untreated net samples, the mean mortality rate was somewhat elevated in the middle of the experimental hut trial period with a peak of 36.0% in January, for both, the washed and the unwashed control nets.

## Discussion

According to WHOPES guidelines [18], a LLIN is expected to retain biological activity (i.e. KD ≥ 95% and/or mortality ≥ 80%) for at least 20 standard WHO washes under laboratory conditions using an *An. gambiae *Kisumu-susceptible strain. Laboratory investigations presented here with ICON^® ^Maxx-treated nets (polyester and polyethylene) revealed considerably lower biological activity well before 20 washings. Indeed, only the impregnated polyethylene net was above the WHOPES threshold with a mean KD of 98.0% prior to the first washing. After five washings, however, the mean KD dropped below the 95% benchmark. All other bioassays with the Kisumu-susceptible strain showed an efficacy below 95% KD and below 80% mortality. Efficacy gradually decreased with the number of washings. Interestingly though, the dynamics of mortality showed a slight recovery of efficacy, but after five and 10 washings, ICON^® ^Maxx activity decreased continuously for both net types. The current results are in contrast to findings observed in previous WHO trials where ICON^® ^Maxx met established efficacy criteria [16].

What are possible explanations for the low efficacy of ICON^® ^Maxx observed in the laboratory in the present study? A first explanation could be variability in insecticide loading following net treatment. It is conceivable that squares of netting sampled for bioassay did not contain the full, recommended dose of lambda-cyhalothrin and as such demonstrated reduced efficacy in the laboratory. Whilst the impact of this may be seen in the laboratory, it is unlikely to have an effect under field conditions where an effective dose may be acquired by a mosquito given that mosquitoes may search and contact large areas of a treated net in search of a blood meal [29].

Another factor to be considered is the repellent effect of pyrethroids on mosquitoes, which is widely acknowledged and has previously been confirmed in a hut trial in which the total number of mosquitoes caught in control huts was much higher than in the test huts [30]. Indeed, the repellent effect is pronounced in the WHO cone test, as a large surface area within the cone is not covered by the net, and hence mosquitoes may not be in contact with the insecticides for the whole 3 min period. This hypothesis is supported by studies on another pyrethroid-treated netting material, PermaNet 2.0^® ^(Vestergaard-Fraansen), where 20 standard washes revealed mortality of 100% using WHO cylinders [16] but lower values (81.8-87.1%) when using WHO cones [6,31]. In view of these results, data derived from multicentre trials comparing cones and cylinders for net bioassays may shed new light on this issue. While the use of cones is standard to WHOPES guidelines [18], the use of ball/wire frames or cylinders is a deviation of the recommended WHOPES phase I evaluation procedure. The current results call for a review of existing WHOPES evaluation procedures.

The subsequent 12-month experimental hut trial in central Côte d'Ivoire showed that, despite the high levels of pyrethroid resistance in the local malaria vectors [19,20], ICON^® ^Maxx yielded high rates of mosquito deterrence, induced exophily and reduced blood-feeding against free-flying mosquito populations. Baseline cone bioassays showed KD and mortality rates close to the WHOPES cut-off values (94.3% and 78.1%, respectively). Although both KD and mortality rates somewhat decreased within the first six months of the trial, both measures were consistently above the WHOPES predefined thresholds in the second half of the trial after half of the nets were washed once (to mimic the local context as nets are usually washed every six months). These entomological features are likely to have a positive impact in reducing malaria transmission [32].

A crucial point for consideration of reduced mortality in the experimental hut trial is pyrethroid resistance of the local *An. gambiae *mosquito population as confirmed in the bioassays, and further results from a recent study carried out in the same site [19] and another experimental hut setting in close proximity [20]. Additional studies have been launched to fully characterize this pyrethroid-resistant *An. gambiae s.s*. field population, by pursuing gene expression studies.

## Conclusion

In the current study, ICON^® ^Maxx-treated nets did not meet WHOPES cut-off criteria under laboratory conditions, and hence the findings reported here are in contrast to previous results [16]. However, under semi-natural conditions, nets treated with ICON^® ^Maxx yielded high rates of mosquito deterrence, induced exophily and mortality, and reduced blood-feeding despite a high level of pyrethroid resistance in the local *An. gambiae *population. Hence, it is conceivable that nets treated with ICON^® ^Maxx considerably impact on malaria transmission. The present findings highlight the need for field evaluations, including detailed insecticide resistance characterisation of the local mosquito fauna. Standardized laboratory test methods warrant continues monitoring and evaluation.

## Competing interests

This study received financial support from Syngenta Crop Protection. The study design, data collection and analysis, decision to publish and preparation of the manuscript was under the responsibility of the scientists from the different research institutions. All authors declare that they have no conflict of interest. MSW, ET and JU are the guarantors of the paper.

## Authors' contributions

MSW carried out the laboratory investigation and drafted the manuscript. ET and JD carried out the experimental hut trial, performed the pyrethroid resistance testing, conducted the statistical analysis and assisted in drafting the manuscript. BGK conceived the study design, coordinated the laboratory investigation and assisted with the statistical analysis. CN coordinated the experimental hut trial and assisted with the data analysis. PM analysed the field data and assisted with the manuscript revision. AMA coordinated the laboratory investigation and logistics for the experimental hut trial. JU was the overall study coordinator and contributed to the interpretation of the data and manuscript writing and revision. All authors read and approved the final manuscript.
